# Changes of Serum Bone Metabolism Indexes and Ultrasonic Bone Mineral Density in Patients with Diabetic Nephropathy at Different Stages and their effects on Diabetic Renal Microvascular Complications

**DOI:** 10.12669/pjms.39.3.6650

**Published:** 2023

**Authors:** Lulu Han, Shenghai Wang, Jingjing Ma, Ningning Song, Zhao Wang, Mingyan Yao

**Affiliations:** 1 Lulu Han, Department of Endocrinology, Baoding No.1 Central Hospital, Baoding 071000, Hebei, China; 2 Shenghai Wang, Intensive Care Unit, Affiliated Hospital of Hebei University, Baoding 071000, Hebei, China; 3 Jingjing Ma, Department of Endocrinology, Baoding No.1 Central Hospital, Baoding 071000, Hebei, China; 4 Ningning Song, Department of Endocrinology, Baoding No.1 Central Hospital, Baoding 071000, Hebei, China; 5 Zhao Wang, Department of Endocrinology, Baoding No.1 Central Hospital, Baoding 071000, Hebei, China; 6 Mingyan Yao Department of Endocrinology, Baoding No.1 Central Hospital, Baoding 071000, Hebei, China

**Keywords:** Diabetic Nephropathy, Bone Metabolism Index, Ultrasonic Bone Mineral Density

## Abstract

**Objective:**

To determine the changes in serum bone metabolism indexes and ultrasonic bone mineral density (BMD) in patients with diabetic nephropathy at different stages, and their effects on diabetic renal microvascular complications.

**Methods:**

This is a clinical comparative study. One hundred twenty two diabetic patients admitted to the Baoding No.1 Central Hospital from January 2020 to March 2022 were selected as subjects and divided into three groups according to their actual condition: the simple diabetes (Group-A, 40 cases), diabetic nephropathy with micro urinary protein (Group-B, 40 cases) and diabetic nephropathy with massive proteinuria (Group-C, 42 cases). Another 36 healthy subjects were selected as the control group. Differences in serum bone metabolism indexes and ultrasound BMD levels were compared.

**Results:**

Twenty five hydroxy-vitamin D, BGP, T-PINP and ultrasound BMD levels in the control group were > Group-A > Group-B > Group-C, PTH and β-CTX in the control group were < Group-A < Group-B < Group-C, statistically significant differences (p<0.05). The urinary albumin to urinary creatinine ratio (ACR) value in Group-B was significantly lower than Group-C (p<0.05). Logistic regression analysis showed that 25-hydroxy-vitamin D, PTH, BGP, β-CTX, T-PINP and ultrasound BMD were the influencing factors of diabetic renal microvascular complications (p<0.05).

**Conclusion:**

Bone metabolism indexes and ultrasound bone mineral density are abnormally expressed in patients with diabetic nephropathy at different stages, which are closely related to the urine protein of patients. They have important clinical value in the diagnosis of early diabetic nephropathy.

## INTRODUCTION

Diabetes is one of the important factors leading to cardiovascular and cerebrovascular diseases and renal failure in the body.^1^ Diabetic nephropathy, as one of the common microvascular complications of diabetes, specifically refers to chronic kidney disease caused by diabetes. Early diabetic nephropathy is mostly manifested by increased excretion rate of microalbumin in the urine, and decreased glomerular filtration rate can be seen in renal function examination with the aggravation of the disease.^2^ Those with severe disease may involve dialysis and kidney transplantation, and will have a significantly increased probability of cardiovascular disease along with the decline of renal function, which will seriously endanger life safety.^3^ Studies have revealed that there is a correlation between nephropathy and abnormal changes in bone metabolism indicators in the body. For patients with diabetic nephropathy, their body will have an imbalance of calcium and phosphorus metabolism due to the excretion of a large amount of albumin in the urine, resulting in osteoporosis.^4^ In addition, they have a significantly higher incidence of bone loss and osteoporosis compared with the same age group.^5^ Therefore, in this study, the changes in serum bone metabolism indexes and ultrasound BMD in patients with diabetic nephropathy at different stages and their effects on diabetic renal microvascular complications were investigated, in order to predict the risk of diabetic renal microvascular complications early in clinical practice, and to provide theoretical support for early intervention and delay of progression of diabetic nephropathy.

## METHODS

This is a clinical comparative study. A total of 122 diabetic patients admitted to the Second Department of Endocrinology, Baoding No.1 Central Hospital from January 2020 to March 2022 were selected as subjects, and were divided into the simple diabetes group (Group-A, ACR<30 mg/g, 40 cases) and the diabetic nephropathy group (82 cases) according to whether they are complicated with diabetic renal microvascular complications. The study was approved by the Institutional Ethics Committee of Baoding No.1 Central Hospital(No.:2022004; Date: February 16, 2022), and written informed consent was obtained from all participants.

Patients in the diabetic nephropathy group were divided into diabetic nephropathy patients with micro urinary protein (Group-B, 30 mg/g<ACR<300 mg/g, 40 cases) and diabetic nephropathy patients with massive proteinuria (Group-C, 300 mg/g<ACR, 42 cases) according to the degree of urinary albumin to urinary creatinine ratio (ACR). Another 36 healthy subjects who went to Baoding No.1 Central Hospital for routine physical examination during the same period were selected as the control group. There were no statistical differences in sex ratio and average age among the four groups (p>0.05).

### Inclusion criteria:

- Patients with diabetes and diabetic nephropathy clearly diagnosed in hospital and meet the relevant diagnostic criteria of Guideline for the Prevention and Treatment of Type-2 Diabetes Mellitus in China (2020 edition) and Clinical Guideline for the Prevention and Treatment of Diabetic Kidney Disease in China (2021 edition).^6^
- Patients aged from 18-80 years.
- Patients with clear in-hospital examination results and complete clinical data.
- Patients who knew the content of the study, were aware of the advantages and disadvantages, and had signed informed consent.

### Exclusion criteria:

- Patients with Type-1 diabetes, gestational diabetes and other special types of diabetes.
- Patients with thyroid disease, parathyroid disease and primary osteoporosis.
- Patients with severe liver and kidney dysfunction, coagulopathy and severe mental illness.
- Patients with autoimmune diseases, nephropathy, tumors and other diseases that affect bone metabolism.
- Patients who have used drugs that affect bone metabolism in the past six months, such as bisphosphonates and glucocorticoids.

### Methods:

(1) Measurement of bone metabolism indicators: 5ml of venous blood was collected on an empty stomach, and 25-hydroxy-vitamin D, parathyroid hormone (PTH), and osteocalcin (BGP) in patients were detected by direct chemiluminescence. Electrochemiluminescence was utilized to detect the total Type-I collagen amino-terminal extension peptide (T-PINP) and the Type-I collagen carboxyl-terminal peptide β special sequence (β-CTX). (2) Glucose metabolism indicators: HbA1c, Serum insulin (INS) and C-peptide (C-peptide). (3) Biochemical indicators: fasting blood glucose, blood calcium, blood phosphorus; (4) Urine indicators: 3ml urine samples were collected on an empty stomach. Microalbumin was determined by immunoturbidimetry, and the ACR was calculated. (5) Measurement of ultrasonic bone density: The bone mineral density of the patients was measured by ultrasonic bone density tester.

### Statistical processing:

All data were analyzed by SPSS 22.0 statistical software. Enumeration data were expressed by (n, %) and tested by *χ^2^*. The measurement data subject was represented by (*χ̅*±*S*), one-way ANOVA analysis of variance was performed, and LSD was used for pairwise comparisons afterwards. Pearson correlation analysis was used for correlation analysis. Moreover, binary logistic regression was utilized to analyze the independent risk factors of diabetic renal microvascular complications. P<0.05 indicates a statistically significant difference.

## RESULTS

The fasting blood glucose, blood urea nitrogen, and glycation in the control group were lower than those in groups A, B, and C, with statistically significant differences (p<0.05), see Table-I. The ACR value of the control group was significantly lower than that of Groups B and C, while Group-B was significantly lower than that of Group-C (p<0.05), see Table-II.

**Table-I:** Comparison of general indicators among the four groups (*χ̅*±*S*).

Group	Number of cases	Fasting blood glucose (mmol/L)	Creatinine (μmol/L)	Urea nitrogen (mmol/L)	Saccharification (mmol/L)	Insulin (uIU/L)	C-peptide (ng/ml)	Calcium (mmol/L)	Phosphorus (mmol/L)
Control group	36	5.29±0.35	53.90±11.53	4.52±1.25	5.34±0.25	9.81±2.73	1.95±0.60	2.35±0.12	1.23±0.21
Group-A	40	8.99±2.57^a^	62.05±12.14^a^	5.55±1.19^a^	7.87±1.71^a^	13.40±17.50	2.18±0.80	2.39±0.47	1.30±0.17
Group-B	40	9.06±2.60^a^	64.21±13.33^a^	5.34±1.32^a^	8.45±1.87^a^	10.72±6.50	2.21±0.89	2.31±0.11	1.35±0.22^a^
Group-C	42	9.81±2.61^a^	75.71±18.13^abc^	5.62±1.66^a^	8.46±1.45^a^	18.77±11.47^abc^	3.12±1.40^abc^	2.28±0.11	1.37±0.22^a^
F	-	29.488	15.942	5.017	37.386	5.234	11.134	1.475	3.216
p	-	<0.001	<0.001	0.002	<0.001	0.002	<0.001	0.223	0.025

***Note:***

a p<0.05 compared with the control group; ^b^p<0.05 compared with group A; ^c^p<0.05 compared with Group-B.

**Table-II:** Comparison of ACR values among the four groups (*χ̅*±*S*).

Group	Number of cases	ACR value
Control group	36	7.58±3.81
Group-A	40	11.88±6.05
Group-B	40	82.64±49.89^ab^
Group-C	42	634.23±306.54^abc^
F	-	145.669
p	-	<0.001

***Note:*** ^a^p<0.05 compared with the control group; ^b^p<0.05 compared with Group-A; ^c^p<0.05 compared with Group-B.

T-PINP in the control group > Group-A > Group-B > Group-C, while PTH and β-CTX in the control group < Group-A < Group-B < Group-C, with a statistically significant difference (p<0.05), see Table-III.

**Table-III:** Comparison of bone metabolism indexes among the four groups (*χ̅*±*S*).

Group	Number of cases	25-hydroxy-vitamin D (ng/ml)	PTH (pg/ml)	BGP (ng/ml)	β-CTX (ng/ml)	T-PINP (ng/ml)
Control group	36	33.03±5.83	23.08±8.32	32.05±9.52	0.65±0.39	82.69±57.63
Group-A	40	21.60±4.68^a^	37.64±11.68^a^	24.22±7.46^a^	1.04±0.54^a^	37.06±13.24^a^
Group-B	40	13.73±3.36^ab^	54.80±17.20^ab^	15.36±5.84^ab^	1.79±0.63^ab^	22.98±7.45^ab^
Group-C	42	9.87±3.41^abc^	98.18±25.47^abc^	9.91±5.19^abc^	4.89±2.86^abc^	17.40±7.27^abc^
F	-	207.181	142.118	73.098	62.402	39.564
p	-	<0.001	<0.001	<0.001	<0.001	<0.001

***Note:***

a p<0.05 compared with the control group; ^b^p<0.05 compared with Group-A; ^c^p<0.05 compared with Group-B.

**Fig.1:**
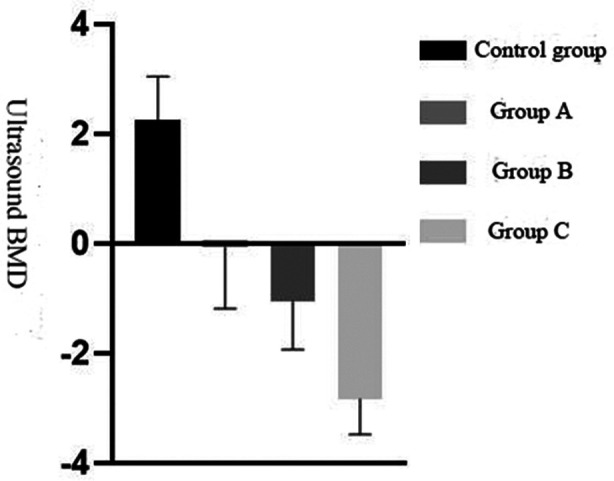
Comparison of ultrasound bone mineral density among the four groups.

As for the comparison of ultrasound BMD among the four groups (*χ^2^*=127.992, p<0.001), that of the control group was > Group-A > Group-B > Group-C, with a statistically significant difference (p<0.05), see Fig.1.

**Fig.2:**
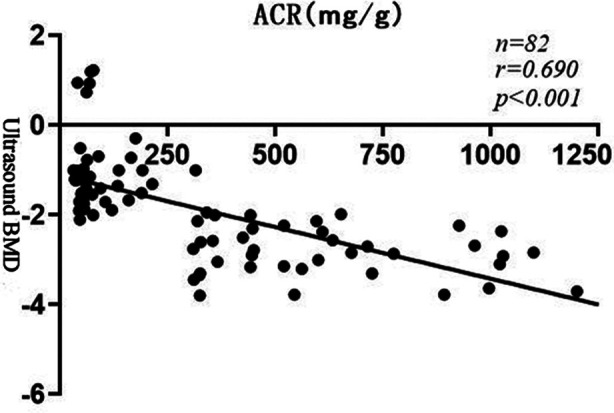
Correlation analysis between ACR and BMD.

ACR was negatively correlated with vitamin D, BGP, T-PINP, and positively correlated with PTH, β-CTX, creatinine, insulin, and C-peptide; Ultrasound BMD was negatively correlated with PTH, β-CTX, creatinine, insulin, and C-peptide, and positive correlation with vitamin D, BGP, T-PINP, and calcium, see Table-IV; Moreover, ACR was negatively correlated with BMD (r=-0.690, p<0.001), see Fig.2.

**Table-IV:** Correlation analysis of bone metabolism indexes, BMD and ACR in patients with diabetic nephropathy.

Indexes	ACR	BMD	ACR	BMD

	r	p	r	p
Vitamin D	-0.493	<0.001	0.424	<0.001
PTH	0.622	<0.001	-0.553	<0.001
BGP	-0.377	<0.001	0.340	<0.001
T-PINP	-0.357	0.001	0.467	<0.001
β-CTX	0.625	<0.001	-0.528	<0.001
Fasting blood sugar	0.091	0.415	0.009	0.937
Creatinine	0.343	0.002	-0.305	0.005
Urea nitrogen	0.158	0.156	0.006	0.957
Saccharification	0.036	0.745	0.097	0.384
Insulin	0.330	0.002	-0.404	<0.001
C-peptide	0.423	<0.001	-0.464	<0.001
Calcium	-0.150	0.177	0.243	0.028
Phosphorus	-0.018	0.873	0.063	0.573

**Fig.3:**
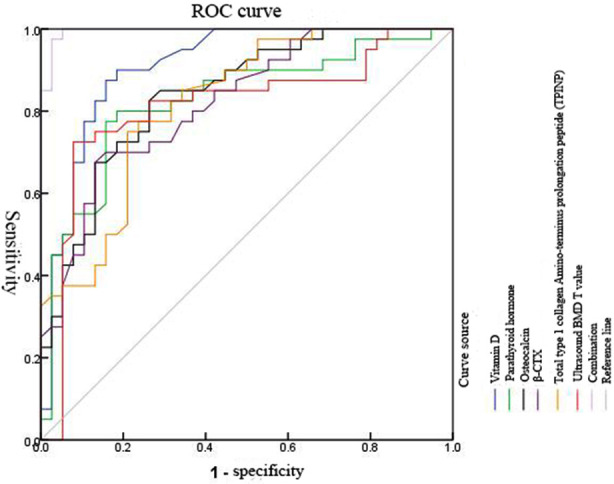
ROC curve of bone metabolism index and ultrasound BMD for diagnosis of early diabetic nephropathy.

Logistic regression analysis showed that vitamin D, PTH, BGP, β-CTX, T-PINP and ultrasound BMD were the influencing factors of diabetic renal microvascular complications (p<0.05), see Table-V. The area under the curve (AUC) of 25-hydroxy-vitamin D, PTH, BGP, β-CTX, T-PINP and ultrasound BMD were 0.910, 0.826, 0.841, 0.822, 0.821, 0.811 respectively in the diagnosis of early diabetic nephropathy, and the AUC of combined diagnosis was 0.995. See Table-VI and Fig.3.

**Table-V:** Logistic regression analysis of factors affecting diabetic nephropathy.

Factors	β	Standard Error S.E.	Wald value	p value	OR value	95% CI
25-hydroxy-vitamin D	-0.508	0.094	29.197	0.000	0.602	0.501~0.724
PTH	0.106	0.022	23.401	0.000	1.112	1.065~1.161
BGP	-0.329	0.064	26.715	0.000	0.720	0.635~0.815
T-PINP	-0.174	0.033	27.889	0.000	0.840	0.787~0.896
β-CTX	2.524	0.516	23.882	0.000	12.476	4.534~34.330
Ultrasound BMD	-1.323	0.251	27.743	0.000	0.266	0.163~0.436
Constant term	-0.614	0.333	3.399	0.065	0.541	/

**Table-VI:** ROC curve analysis of bone metabolism indexes and ultrasound BMD in the diagnosis of early diabetic nephropathy.

Test outcome variables	AUC	SE	p	Youden index	Cut-off value	Sensitivity	Specificity	95%Cl
25-hydroxy-vitamin D	0.910	0.035	0.000	0.717	18.005	0.875	0.842	0.842~0.977
PTH	0.826	0.049	0.000	0.617	44.36	0.775	0.842	0.731~0.921
BGP	0.841	0.044	0.000	0.562	19.800	0.825	0.737	0.754~0.927
β-CTX	0.822	0.046	0.000	0.543	1.650	0.675	0.868	0.731~0.912
T-PINP	0.821	0.047	0.000	0.539	26.935	0.750	0.789	0.729~0.913
Ultrasound BMD	0.811	0.053	0.000	0.646	0.995	0.725	0.921	0.706~0.915
Combination	0.995	0.005	0.000	0.949	/	0.975	0.974	0.986~1.000

## DISCUSSION

In this study, the levels of vitamin D, osteocalcin, T-PINP and ultrasound bone mineral density decreased with the increase of proteinuria, while the levels of PTH and β-CTX increased with the increase of proteinuria. Correlation analysis showed that ACR was negatively correlated with vitamin D, BGP, T-PINP, and BMD, and positively correlated with PTH and β-CTX. This shows that vitamin D, BGP, T-PINP and BMD decrease with the increase of urine protein in patients with diabetes, while PTH and β-CTX increase with the increase of urine protein. Yang Xiong et al. revealed that the degree of mineral bone metabolism disorder in patients with stages three to five chronic kidney disease would gradually worsen with the increase of stage.^7^

Diabetic nephropathy, as a common microvascular complication of diabetes, has a complex etiology and lacks sensitive early warning indicators in the early stage of the disease. Epidemiological survey results show that the incidence of diabetic nephropathy accounts for about 40% of diabetic patients. This disease is highly insidious at the onset,^8^,^9^ and there is a lack of detection methods for specificity and sensitivity. Patients with early diabetic nephropathy have no significant symptoms, with or without positive microalbuminuria. As the disease progresses, typical characteristics such as hypertension, edema, anemia and fatigue may occur. At this time, patients often have a lot of proteinuria or renal insufficiency.^10^

Several studies have pointed out that diabetic nephropathy has a close bearing on osteoporosis, in addition, diabetic microangiopathy may lead to oxygen supply and blood supply disorders to bone tissue, which further aggravates the condition of such patients.^11^ Patients with diabetic nephropathy are in a state of chronic hypoxia due to long-term high blood sugar levels. Microcirculation disorders make it impossible for the body to ensure the supply of blood and nutrients to bones in a timely manner, leading to osteoporosis.^12^ Besides, hyperglycemia osmotic diuresis action itself promotes the rapid loss of electrolytes, such as blood calcium, phosphorus, and magnesium. As a result, the bone balance is broken, which accelerates the occurrence of osteoporosis. 25-hydroxy-vitamin D in the human body is synthesized by sun irradiation on the skin and is hydroxylated in the liver and kidney to form 1,25 (OH)2D. The kidney is the target organ of vitamin D, and its receptors are highly expressed in renal cells, such as popocytes, mesangial cells and renal tubule collecting system. Consequently, patients with diabetic kidney injury are often associated with vitamin D deficiency and varying degrees of bone loss. As a protein containing γ-carboxyglutamate, the level of BGP can directly reflect the activity of osteoblasts and has a positive correlation with the bone calcium content. When the concentration of extracellular calcium ions decreases, the amount of PTH secreted by the parathyroid glands will increase.^13^,^14^ T-PINP is a specific marker of new bone formation, and its serum content can reflect the synthesis of Type-I collagen and the situation of bone formation and turnover. β-CTX, as an expression of the carboxy-terminal peptide of Type-I collagen, can reflect osteoclast activity.^15^ In the early stage of diabetic nephropathy, especially when there is no significant change in BMD, the bone synthesis indexes such as BGP and T-PINP will decrease, and the level of osteolysis index β-CTX will increase. To this end, bone metabolism markers can reflect the dynamic changes in bone metabolism in patients with diabetic nephropathy earlier.^16^,^17^ Previous studies have found that bone metabolism indexes can sensitively reflect the risk of osteoporosis, which are of high accuracy in predicting osteoporosis.^18^ In this study, the analysis of the correlation between bone metabolism indexes, BMD and the progression of urinary protein in diabetic nephropathy shows that the three are closely related. In addition, because the changes in bone metabolism indexes in patients with nephropathy are earlier than changes in BMD, they can be used as sensitive indicators for early detection of the risk of diabetic nephropathy and detection of patients with diabetic nephropathy.^19^,^20^ The conclusion of this study adds clinical reference data for early intervention to prevent progression of diabetic nephropathy.

### Limitations of this study:

There are still some shortcomings in this study. This study was a retrospective descriptive study, with limited clinical data available and limited persuasive conclusions. Further intervention trials are needed in the future to confirm these results.

## CONCLUSION

To put it in a nutshell, bone metabolism indexes and ultrasound bone mineral density are abnormally expressed in patients with diabetic nephropathy at different stages, which are closely related to the urine protein of patients. In this study, 25-hydroxyvitamin D, BGP, β-CTX, PTH, T-PINP, and ultrasound BMD are combined to diagnose early diabetic nephropathy, which is of great clinical value.

### Authors’ Contributions:

**LH and SW** designed this study and prepared this manuscript, and are responsible and accountable for the accuracy or integrity of the work.

**JM and NS** collected and analyzed clinical data.

**ZW and MY** significantly revised this manuscript.
